# Intragenic Recombination Has a Critical Role on the Evolution of *Legionella pneumophila* Virulence-Related Effector *sidJ*

**DOI:** 10.1371/journal.pone.0109840

**Published:** 2014-10-09

**Authors:** Joana Costa, Paulo Gonçalves Teixeira, Ana Filipa d'Avó, Célio Santos Júnior, António Veríssimo

**Affiliations:** 1 Department of Life Sciences, University of Coimbra, Coimbra, Portugal; 2 CNC - Center for Neuroscience and Cell Biology, University of Coimbra, Coimbra, Portugal; 3 Department of Chemical and Biological Engineering, Chalmers University of Technology, Gothenburg, Sweden; 4 Department of Molecular Biology and Evolutionary Genetics, Federal University of São Carlos (UFSCar), São Paulo, Brazil; University of Louisville, United States of America

## Abstract

SidJ is a Dot/Icm effector involved in the trafficking or retention of ER-derived vesicles to *Legionella pneumophila* vacuoles whose mutation causes an observable growth defect, both in macrophage and amoeba hosts. Given the crucial role of this effector in *L. pneumophila* virulence we investigated the mechanisms shaping its molecular evolution. The alignment of SidJ sequences revealed several alleles with amino acid variations that may influence the protein properties. The identification of HGT events and the detection of balancing selection operating on *sidJ* evolution emerge as a clear result. Evidence suggests that intragenic recombination is an important strategy in the evolutionary adaptive process playing an active role on *sidJ* genetic plasticity. This pattern of evolution is in accordance with the life style of *L. pneumophila* as a broad host-range pathogen by preventing host-specialization and contributing to the resilience of the species.

## Introduction

*Legionella pneumophila* is a ubiquitous bacterium in freshwater environments as well as in many man-made water systems worldwide known for its ability to cause pneumonia in humans [1]. *L. pneumophila* are subject to predation by eukaryotic phagocytes, such as amoeba and ciliates, so the bacterium's survival and spread depends on the ability to hijack the phagocytic vacuole, to create a replicative niche, to prevent phagosome-lysosome fusion and evade host immune system. In humans, *L. pneumophila* reaches the lungs after inhalation of contaminated aerosol droplets where the similar mechanisms allow *L. pneumophila* to hijack another phagocyte, lung-based macrophages, leading to infection [2]–[10]. Since human-to-human transmission of *L. pneumophila* has not been observed the human infection is an evolutive dead end for *Legionella*. Consequently, protozoan hosts are believed to provide the primary evolutionary pressure for the acquisition and maintenance of virulence factors, resulting largely from the organism's need to replicate in an intracellular niche and also avoid predation by environmental protozoa [4], [5], [8], [10].

The long-term co-evolution of *L. pneumophila* with free-living amoebae has influenced the genomic structure of this organism since amoeba may act as a gene melting pot, allowing diverse microorganisms to evolve by gene acquisition and loss, and then either adapt to the intra-amoebal lifestyle or evolve into new pathogenic forms [8], [10]–[12]. This lifestyle, namely the interaction with different protozoan in different environments, may have prevented host-specialization and be responsible for the evolutionary story of *L. pneumophila* [13]. Several studies showed that *L. pneumophila* clinical isolates showed less genetic diversity than man-made and natural environmental isolates [14]–[19]. This evidence supports the hypothesis proposed by Coscollá and González-Candelas [16] that isolates of *L. pneumophila* recovered from clinical cases are a limited, non-random subset of all genotypes existing in nature, perhaps representing an especially adapted group of clones.

The virulence of *L. pneumophila* is dependent on the Dot/Icm type IVB protein secretion system responsible for the translocation of at least 290 effectors into the host cell where they act on diverse host cell pathways [20]–[22]. Functional redundancy among groups of substrates that target similar host processes has been commonly reported since elimination of a single substrate gene rarely leads to detectable defects in intracellular growth under standard laboratory conditions [3]–[5], [23]. Indeed, its particular large repertoire of effectors seems to be the basis for the broad host range of *L. pneumophila*, since replication within different hosts requires specific sets of substrates [23], [24]. Inter-domain horizontal gene transfer from eukaryotes and subsequent evolution of eukaryotic-like translocated effectors has enabled *L. pneumophila* to adapt to the intracellular lifestyle through exploitation of evolutionarily conserved eukaryotic cell mechanisms [3], [12] Indeed, many of the *dot/icm* effectors harbor eukaryotic-like motifs that mediate the interaction with host proteins and organelles to modulate host cell functions, establishing molecular mimicry as a major virulence strategy in *L. pneumophila* pathogenesis [5], [21], [24]. Although the vast majority of individual Dot/Icm-secreted substrates are genetically dispensable for the intracellular replication of *L. pneumophila*, critical components for both intracellular growth and disease within animals have been identified. Indeed, only SdhA, SidJ and AnkB have been described as essential for maximal intracellular replication, suggesting that certain proteins in *L. pneumophila* selectively provide an advantage to the pathogen in certain hosts [3], [4], [20], [25]–[28]. Furthermore, both *sdhA* and *sidJ* are conserved among strains of *Legionella pneumophila* and *Legionella longbeachae* of known genome sequence [29]–[32].

SidJ modulates host cellular pathways through the membrane remodeling of the *L. pneumophila* containing vacuoles by the efficient acquisition of ER specific proteins [4], [27]. The SidJ locus is presented in an operon-like structure with three other members of the SidE family, namely, *sdeC*, *sdeB* and *sdeA* [29]–[32]. Nevertheless, SidJ clearly is the sole protein responsible for the growth defect observed in the *sidJ* mutant since neither of those genes is required for intracellular growth in macrophages [33], [34]. Moreover, *sidJ* expression is not coregulated by the same mechanisms that rule the expression of *sdeC*, *sdeA*, and *sdeB* [27], which are significantly induced when *L. pneumophila* enters the postexponential growth phase [33]. Compared to wild-type strains, the *sidJ* deletion mutant did not display any detectable growth defect in AYE broth, but resulted in ∼15-fold reduction in intracellular growth within macrophages, and causes a significant growth defect in amoeba [27]. Given the role of SidJ in establishing successful infections and the diversity of host cells encountered by *L. pneumophila* in nature, it is possible that this gene product is a target for host specialization and adaptive evolution, and that variation in *sidJ* may reflect an increase in the fitness of *L. pneumophila* in certain environments. Our goal was to determine the genetic structure and allelic diversity of *L. pneumophila* populations inferred from *sidJ* gene and to identify the molecular mechanisms operating in the evolution of this virulence-related gene.

The identification of HGT events within *L. pneumophila* and the detection of balancing selection operating on *sidJ* evolution emerge from the present work. Our results indicate that intragenic recombination is favored as a strategy in the evolutionary adaptive process playing an active role in *sidJ* genetic plasticity.

## Materials and Methods

### *L. pneumophila* strains

Thirty two unrelated strains of *L. pneumophila* were selected for complete sequencing of the *sidJ* gene to determine the genetic structure and molecular evolution (Table 1). Strains were selected from several others in order to capture the maximum genetic variability, since they represented the allelic diversity determined in early studies from the complete sequence of *dotA* and type II protein secretion system (T2S) related genes [18], [19]. These also included twelve isolates from 9 sites comprising natural and man-made environments, and seventeen clinical-related *L. pneumophila* type and reference strains, eleven from *L. pneumophila* subsp. *pneumophila*, three *L. pneumophila* subsp. *fraseri* strains and three *L. pneumophila* subsp. *pascullei*. The sequences from eleven *L. pneumophila* subsp. *pneumophila* genome sequenced strains [31], [32], [35]–[39] were also included in this work. Previously published sequences of partial *rpoB* gene from the studied strains were also used for comparison purposes (Table S1).

**Table 1:** *L. pneumophila* unrelated strains, isolated from distinct environments, type and reference strains included in this study and distribution of *L. pneumophila* strains into clusters according with *rpoB* and *sidJ* gene sequences.

Strain designation	Environmental type	Subspecies	Reference of the source	Clusters	
				*rpoB*	*sidJ*
Aço13	Natural	*L. pneumophila* subsp. *pneumophila*	[93]	A	B
Aço20	Natural	*L. pneumophila* subsp. *pneumophila*	[93]	A	A
Agn2	Natural	*L. pneumophila* subsp. *pneumophila*	[18]	A	C
Alf 18	Natural	*L. pneumophila* subsp. *pneumophila*	[94]	A	C
Felg244	Natural	*L. pneumophila* subsp. *pneumophila*	[40]	A	C
Ice27	Natural	*L. pneumophila* subsp. *pneumophila*	[18]	A	B
Ice30	Natural	*L. pneumophila* subsp. *pneumophila*	[18]	A	C
NMex1	Natural	*L. pneumophila* subsp. *pneumophila*	[94]	A	C
NMex49	Natural	*L. pneumophila* subsp. *pneumophila*	[94]	A	A
HL06041035	Man-made	*L. pneumophila* subsp. *pneumophila*	[12]	A	B
IMC23	Man-made	*L. pneumophila* subsp. *pneumophila*	[95]	A	C
LPE059	Man-made	*L. pneumophila* subsp. *pneumophila*	[31]	A	A
Ma36	Man-made	*L. pneumophila* subsp. *pneumophila*	[18]	A	C
Por3	Man-made	*L. pneumophila* subsp. *pneumophila*	[18]	A	B
130b	Clinical-related	*L. pneumophila* subsp. *pneumophila*	[39]	A	D
797-PA-H (ATCC 43130)	Clinical-related	*L. pneumophila* subsp. *pneumophila*	[96]	A	D
Alcoy	Clinical-related	*L. pneumophila* subsp. *pneumophila*	[38]	A	C
ATCC43290	Clinical-related	*L. pneumophila* subsp. *pneumophila*	[30]	A	A
Chicago 2 (ATCC 33215)	Clinical-related	*L. pneumophila* subsp. *pneumophila*	[97]	A	A
Concord 3 (ATCC 35096)	Clinical-related	*L. pneumophila* subsp. *pneumophila*	[98]	A	A
Corby	Clinical-related	*L. pneumophila* subsp. *pneumophila*	[37]	A	C
Lens	Clinical-related	*L. pneumophila* subsp. *pneumophila*	[36]	A	D
Lorraine	Clinical-related	*L. pneumophila* subsp. *pneumophila*	[12]	A	A
Paris	Clinical-related	*L. pneumophila* subsp. *pneumophila*	[36]	A	B
Philadelphia 1 (ATCC 33152^T^)	Clinical-related	*L. pneumophila* subsp. *pneumophila*	[35]	A	A
Thunder Bay	Clinical-related	*L. pneumophila* subsp. *pneumophila*	[32]	A	A
Los Angeles 1(ATCC 33156^T^)	Clinical-related	*L. pneumophila* subsp. *fraseri*	[99]	B	E
Dallas 1E (ATCC 33216)	Clinical-related	*L. pneumophila* subsp. *fraseri*	[98]	B	E
Lansing 3 (ATCC 35251)	Clinical-related	*L. pneumophila* subsp. *fraseri*	[84]	B	A
U8W (ATCC 33737^T^)	Clinical-related	*L. pneumophila* subsp. *pascullei*	[84]	C	E
U7W (ATCC 33736)	Clinical-related	*L. pneumophila* subsp. *pascullei*	[84]	C	E
MICU B (ATCC 33735)	Clinical-related	*L. pneumophila* subsp. *pascullei*	[84]	C	E

### DNA extraction, polymerase chain reaction (PCR), cloning and DNA sequencing

The extraction of genomic DNA from the previously selected *L. pneumophila* strains was carried out as previously described by Costa and colleagues [40]. PCRs were performed to amplify the *sidJ* locus (2625 bp) using the primer sets described in Table S2. In general, PCR was carried out using 150–200 ng DNA, 2.0 mM MgCl_2_, 1X reaction buffer, 0.2 µM each dNTP, 5 pmol each primer, and 1 U Taq polymerase (Invitrogen) in 50 µl reaction volumes with the following PCR profile: 5 min a 95°C; 30 cycles of 95°C, 45 s; 50°C, 45 s; a 72°C, 3 min; 7 min at 72°C. Moreover, in some cases it was necessary to adjust the annealing temperatures for individual strains. The amplified PCR products were detected on 1.0% agarose gels stained with ethidium bromide and were purified for sequencing by using an NZYGelpure extraction kit (NZYTech, Lda., Portugal). To obtain the full–length genes the PCR products were cloned using NZY-A PCR cloning kit (NZYTech, Lda., Portugal) according to the manufacturer instructions. Positive clones were selected on Luria-Bertani agar plates containing 20 µg ml^−1^ X-Gal (5-bromo-4-chloro-3-indolyl-β-D-galactopyranoside), 0.5 mM IPTG (isopropyl-β-D-1-thiogalactopyranoside), and 100 µg ml^−1^ ampicillin. Plates were incubated overnight at 37°C in selective media. Positive clones were confirmed by PCR with the same primers used for amplification, and plasmid DNA was extracted using Zyppy Plasmid Miniprep Kit (Zymo Research, USA) according to the manufacturer instructions. Gene sequences were determined by Macrogen Corporation (Netherlands).

For PCR amplification of the *sdeC*, *laiE*, *sdeB* and *sedA* genes, primers were designed based on the corresponding genes from *L. pneumophila* strain Philadelphia 1, namely, lpg2153, lpg2154, lpg2156 and lpg2157, respectively (Fig. S1 and Table S2). PCR amplifications were performed as previously described. Several annealing temperatures between 40 and 55°C were tested for 1 min. The amplified PCR products were detected and purified as abovementioned. For confirmation purposes, all PCR products were sequenced with the primers used for amplification by Macrogen Corporation (Netherlands).

### Sequence analysis

The quality of the sequences was manually checked using the Sequence Scanner software (https://products.appliedbiosystems.com). Phylogenetic analyses were performed using MEGA5 package [41]. Alignment against the corresponding genes found in eleven genome sequenced *L. pneumophila* strains obtained from the public databases (Table S1), was performed using the multiple alignment CLUSTAL software [42], included on MEGA5 package. For coding loci alignments were performed with the amino acid sequences and gaps were later introduced in the corresponding nucleotide alignments, thus keeping the correct frame for translation. A multiple alignment of amino acid sequences was obtained using ClustalΩ [43] manually corrected where necessary. The MEGA5 package was used to derive the multiple alignments of nucleotide and positions of doubtful homology were removed using Gblocks [44].

Maximum likelihood (ML) phylogenetic trees were obtained for *sidJ* and *rpoB* loci with PhyML 3.0 [45] with HKY +G [46] and TrN +G+I models [47], respectively. The most appropriate model of nucleotide substitution and likelihood scores assessed by TOPALi V2.5 [48] and by jModeltest [49]. The best model was determined by using the Akaike Information Criterion (AIC) [50], [51]. ML phylogenetic analysis was performed for the amino acid alignment by PhyML 3.0 [45] using the JTT +G+F model [52]. The most appropriate model of amino acid substitution and likelihood scores were assessed by ProtTest 2.4 [53]. Supports for the nodes were evaluated by bootstrapping with 1000 pseudoreplicates.

Genetic variability analyses were performed with DnaSP software [54]. Mean non-synonymous mutations among the three groups were compared through one-way analysis of variance (ANOVA) after arcsine square root data transformation to fulfill ANOVA assumptions.

The locations of the variable nucleotide positions were displayed graphically using the programs PSFIND and HAPPLOT written by Dr Thomas S. Whittam and available at the STEC Center website (http://www.shigatox.net/stec/cgi-bin/programs).

### Molecular Evolution

Neighbour-net analysis [55] was performed and converted to a splits graph using the drawing algorithms implemented in SplitsTree4 software – version 4.6 [56]. The neighbour-net method was based on the pairwise distance matrices calculated with the Jukes–Cantor correction [57] of the *sidJ* sequences alignment performed on the MEGA5 package [41].

Intragenic recombination was screened within the aligned sequences using the program RDP3 [58]. This program identifies recombinant sequences and recombination breaking points using several methods. We choose six of them: RDP [59], GENECONV [60], BootScan [61], Maximum Chisquared Test (MaxChi; [62]), CHIMAERA [63] and Sister Scan (SiScan; [64]). The analysis was performed with default settings for the detection methods, a Bonferroni corrected P-value cut-off of 0.05, and a requirement that each potential event had to be detected simultaneously by four or more methods. The breakpoint positions and recombinant sequence(s) inferred for every detected potential recombination event were manually checked and adjusted where necessary using the extensive phylogenetic and recombination signal analysis features available in RDP3.

The GARD method [65] implemented in datamonkey server [66] was also used to search for evidence of phylogenetic incongruence, and to identify the number and location of breakpoints corresponding to recombination events.

### Neutrality tests and positive selection analysis

Tajima's D [67], Fu and Li's D* and F* [68] and Fu's Fs [69] statistics were calculated for testing the mutation neutrality hypothesis [70], as previously described by Coscollá and colleagues [71] and Costa and colleagues [19]. These statistics were calculated with the program DNASP4.0 [54] using a statistical significance level α = 0.025 and applying the false discovery rate [72], [73] to correct for multiple comparisons and 1000 replicates in a coalescent simulation.

Estimates of the number of non-synonymous and synonymous substitutions at each locus (dN/dS) were calculated using the modified Nei–Gojobori method [74] with Jukes-Cantor correction [57] implemented in MEGA5 package [41].

In order to investigate the presence of positively selected codons in *sidJ* locus, the estimates of both positive and purifying selection at each amino acid site were calculated from the ratio of non-synonymous to synonymous substitutions, known as ω, as previously described [18]. Nucleotide sequences alignment from *L. pneumophila* strains were constructed using the MEGA5 package [41] and analyses were conducted using the Selecton version 2.1 software [75], [76]. The significance of the ω scores was obtained by using a Likelihood Ratio Test that compares two nested models: a null model that assumes no selection (M8a) [77] and an alternative model that does (M8) [78].

Four physicochemical properties (volume, polarity, charge and hydrophobicity) were used to characterize the results of amino acid substitutions in comparisons of translated homologous sequences [79], [80]. Corresponding *dG* values were obtained using Miyata's matrix [81] and were calculated per one amino acid substitution so that they would not depend on the rates of nucleotide substitutions per se [82].

### Nucleotide sequence accession numbers

The complete *sidJ* sequences from *L. pneumophila* strains determined in this study were deposited in the EMBL Nucleotide Sequence Database with Accession No. HG531934–HG531954.

## Results and Discussion

### Sequence analysis and genetic structure inferred from *sidJ*

The complete sequence of *sidJ* (2625 bp) was determined from 32 *L. pneumophila* strains (Table 1) to determine the mechanisms shaping this fundamental virulence-related gene evolution. All *L. pneumophila* studied strains yielded the analyzed gene with the expected size.

Sequences from an internal fragment of the *rpoB* gene, previously obtained from the same *L. pneumophila* strains [17], [18], [83], were included in the analysis (Table S1) because the inferred *rpoB* tree agrees with phylogenetic and phenotypic analyses [84]–[86], that allow the separation of the three *L. pneumophila* subspecies.

**Figure 1:**
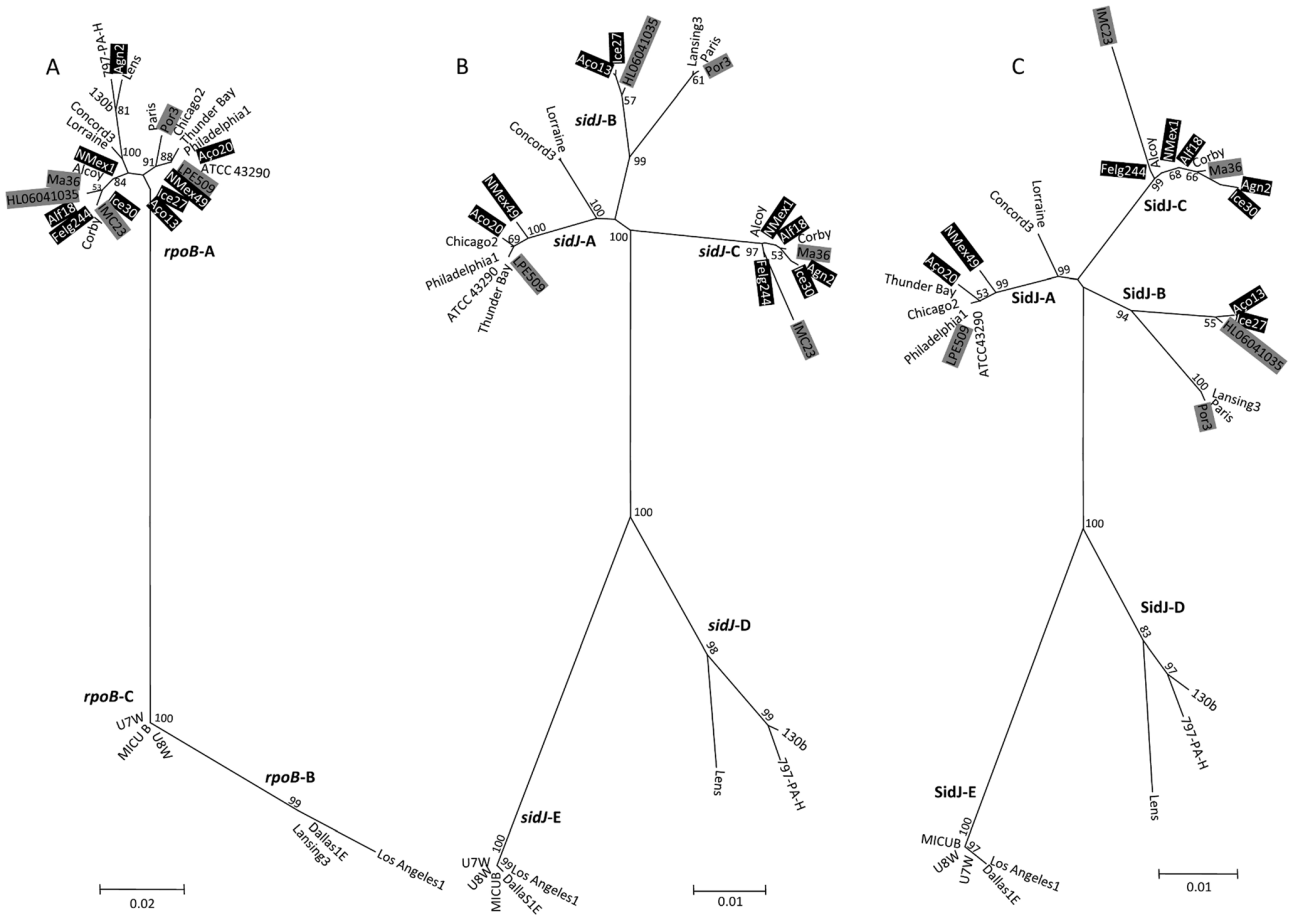
Maximum likelihood phylogenetic trees of *L. pneumophila* isolates, type and references strains (**Table 1**) from DNA sequences of *rpoB* (A), *sidJ* (B) and from deduced amino acid sequences of SidJ (C). Bootstrap support values (1,000 replicates) for nodes higher than 50% are indicated next to the corresponding node.

A comparative analysis between the phylogeny obtained with an internal fragment of *rpoB* gene, used as a marker of vertical inheritance in *L. pneumophila*, and the corresponding phylogeny of *sidJ* was performed to study congruence between this inheritance and the phylogeny of *sidJ*. Maximum likelihood (ML) phylogenetic trees were obtained for *sidJ* and *rpoB* gene sequences (Fig. 1A and B). The topology of the two inferred trees was not congruent since, depending on the gene, most strains had different relationships with each other and with *L. pneumophila* type and reference strains (Fig. 1A and B). The analysis of the *rpoB* gene from the 32 strains matched the three different *L. pneumophila* subspecies, namely, *L. pneumophila* subsp. *pneumophila* (cluster *rpoB*-A), *L. pneumophila* subsp. *fraseri* (cluster *rpoB*-B) and *L. pneumophila* subsp. *pascullei* (cluster *rpoB*-C), comprising 81.2%, 9.4% and 9.4% of all strains, respectively (Fig. 1A and Table 1). While the inferred *rpoB* tree agrees with phylogenetic and taxonomic analyses [80]–[82] with three clusters matching *L. pneumophila* subsp., in the inferred *sidJ* tree five major clusters were identified supported by very high bootstrap values (cluster A to E) (Fig. 1B). One important observation from this study is that the strains previously grouped in the *rpoB*-A cluster (Fig. 1A) (*L. pneumophila* subsp. *pneumophila*) were split into four discrete groups in the *sidJ* sequence-based analysis (cluster A to D) (Fig. 1B). Equally relevant is the fact that the majority of the strains previously clustered in the *rpoB*-B and *rpoB*-C clusters (Fig. 1A) (*L. pneumophila* subsp. *fraseri* and *L. pneumophila* subsp. *pascullei*, respectively) were merged into a single group in the *sidJ* inferred dendrogram (cluster *sidJ*-E) (Fig. 1B). A similar significant evolutionary drift was observed for the strain Lansing 3, that belonged to cluster *rpoB*-B with all other *L. pneumophila* subsp. *fraseri* strains, since it was grouped in a distinct cluster in the ML tree inferred from the *sidJ* gene (*sidJ*-A) along with other *L. pneumophila* subsp. *pneumophila* strains (Table 1). These incongruencies are discussed below in the context of intragenic recombination. Moreover, the strains were not evenly distributed in these clusters. Natural and man-made environmental isolates were only found in clusters *sidJ*-A to C, while clusters *sidJ*-D and *sidJ*-E were composed exclusively by clinical-related strains (Table 1).

Additionally, a phylogenetic comparison between the previously obtained clusters from *rpoB* and *sidJ* genes and the corresponding deduced amino acid sequences was also performed. The ML phylogenetic tree was obtained for SidJ (Fig. 1C). The deduced amino acid sequences from the partial *rpoB* gene sequences of all isolates and reference strains were the same, despite the nucleotide differences detected (results not shown). On the other hand, the clusters inferred from the partial deduced amino acid sequences of s*idJ* (Fig. 1C) were consistent with the previously obtained nucleotide-based subgroups. These findings indicate that most *sidJ* nucleotide polymorphisms result in amino acid changes, in contrast to what was observed for *rpoB* [18]. Moreover, incongruence between lineage relationships was observed for *sidJ* clusters A to C when compared to the nucleotide-based tree (Fig. 1B and C).

### Genetic variability of *sidJ* gene

The overall nucleotide sequence diversity of *rpoB* varied from 0 to 0.032 with an average of 0.043±0.006. (Table S3). The diversity of *sidJ* nucleotide sequences from the five defined clusters was higher than that observed for *rpoB* sequences, varying between 0 and 0.070 with an average of 0.033±0.003. The *sidJ*-B subgroup was the most polymorphic with genetic pairwise differences varying from 0 to 0.026 with an average of 0.015±0.003 (Table S3). On the other hand, the diversity within *sidJ*-D and *sidJ*-E clusters was rather lower. The diversity within the two most representative clusters, *sidJ*-A and *sidJ*-C, varied between 0 and 0.022 with an average of 0.010±0.004 and between 0 and 0.013 with an average of 0.004±0.001, respectively.

Genetic variability of 32 *L. pneumophila* unrelated strains was estimated based on the *sidJ* sequences using genetic diversity parameters, not directly dependent on sample size. Moreover, the genetic variability of *L. pneumophila* populations based on strain origin was also estimated from *sidJ* from natural environmental strains, man-made environmental strains and clinical-related strains (Table 2). The highest haplotype (*h*) was found in clinical-related strains presenting 13 distinct alleles. On the contrary, the haplotype diversity (*Hd*) was higher in natural and man-made environmental isolates since all strains were different from each other. The nucleotide diversities (*π*), number of polymorphic nucleotide sites (*S*), population mutation ration (*θ*), average number of pairwise nucleotide differences (*k*), and total number of mutations (*η*) were higher in clinical-related strains. Non-synonymous mutations were more frequent in man-made environmental strains (39.26%, 68 of 184), while in clinical-related strains and natural populations, mutations accounting for differences among alleles accounted for 32.20% (123 of 382) and 35.33% (53 of 153), respectively. Nevertheless, these differences were not significant among the three populations (F_2,29_ = 3.11; p = 0.06). The overall degree of variability detected within *sidJ* is similar to that previously observed for the *pilD* gene, a structural component of the T2S involved in virulence-related phenotypes found to be under neutral evolution [19], [87].

**Table 2:** Summary of genetic diversity parameters for *sidJ* from *L. pneumophila* strains.

	*sidJ*			
	Overall	Natural environment	Man-made environment	Disease-related
Sequence, *n*	32	9	5	18
Sequence length, *L*	2628	2628	2628	2628
Haplotypes, *h*	23	9	5	13
Haplotype diversity, *Hd*	0.974	**1.0**	**1.0**	0.954
(standard deviation)	(0.015)	**(0.057)**	**(0.126)**	(0.034)
Nucleotide diversity, *π*	0.04778	0.02459	0.03356	**0.05616**
(standard deviation)	(0.00475)	(0.00408)	(0.00521)	**(0.00393)**
Polymorphic sites, *S* (%)	432 (16.43)	151 (17.24)	181 (20.66)	**385 (43.95)**
*θ* (φρομ *Σ*)	0.04096	0.02117	0.03317	**0.04274**
(standard deviation)	(0.01246)	(0.00172)	(0.01662)	**(0.01484)**
Pairwise differences, *k*	125.145	64.556	87.900	**147.092**
Total number of mutations, *η*	424	153	184	**382**
Synonymous mutations (%)	275 (64.86)	97 (64.67)	109 (59.24)	**259 (67.80)**
Non-synonymous mutations (%)	149 (35.14)	53 (35.33)	**68 (39.26)**	123 (32.20)
d*N*/d*S*	0.125	0.142	**0.170**	0.120
*dG* per one amino acid change	1.35	**1.41**	1.37	0.98

The rates of non-synonymous substitutions per non-synonymous site (*dN*) in the coding loci were very low, despite the relatively large values of polymorphic sites, most of which corresponded to synonymous substitutions (*dS*), ranged between 0.081 in natural isolates to 0.2257 in clinical-related strains. The low *dN/dS* ratios obtained for *sidJ* and for the *sidJ*-related populations indicated that these alleles were under purifying selection (Table 2). In this case, variation occurs only if it does not confer a significant disadvantage on any surviving variant. Because nucleotide substitutions may exert their influence on the function of the final protein product at any of several levels (e.g. DNA, mRNA or protein), *dN/dS* ratios reflect general restrictions on gene and protein variability. On the other hand, *dG* values reflect variation purely in protein structural and functional features, indicating some restrictions on the amino acid substitutions at the level of the final functioning product [82]. Based on this analysis we can conclude that the high calculated *dG* values for the *sidJ* and for all *sidJ*-related populations indicates that some of the amino acid substitutions may influence protein properties (Table 2). In fact, despite displaying relatively low *dN/dS* values, not all amino acid substitutions are conservative, as assessed by changes in amino acid physicochemical properties.

### *L. pneumophila* phylogeny inferred from *sidJ* sequences

**Figure 2:**
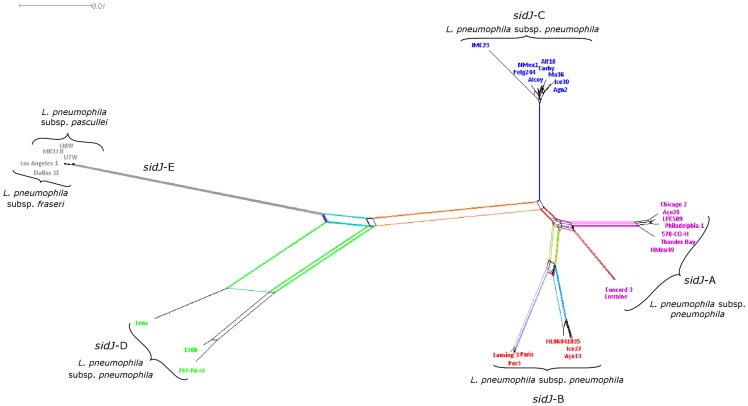
Neighbor-net phylogenetic network showing the relationships among *L. pneumophila* strains (see **Table 1**). The split graph was estimated with SplitsTree4 from *p*-distances of the *sidJ* sequence alignment based on the Jukes–Cantor method. Color code: *sidJ*-A subgroup is shown in purple, *sidJ*-B red, *sidJ*-C blue, *sidJ*-D green and *sidJ*-E grey. The relations between and within strains are illustrated by weighted splits with different colors representing simultaneously both grouping in the data and evolutionary distances between taxa, highlighting conflicting signals or alternative phylogenetic histories (recombination or gene transfer) in *sidJ* molecular evolution.

Neighbor-Net analysis [55] has been performed to determine how recombination and horizontal gene transfer events affected the phylogenetic relationships among *L. pneumophila* strains isolated from distinct environments and locations inferred from *sidJ* sequences (Fig. 2). The obtained splits graph showed evidence of a network-like evolution, indicating the lack of tree-like relationship between the *sidJ* sequences (Fig. 2), although it was still possible to reconstruct the previously defined clusters by the ML phylogenetic analysis (Fig. 1). The center of the neighbor net was slightly netted, implying that the data supports many deep conflicting splits. Nonetheless, the clusters previously identified were quite robust (as indicated by the colors in Figure 2) and the divergence of clusters *sidJ*-A, *sidJ*-B and *sidJ*-C from clusters *sidJ*-D and *sidJ*-E was noticeable. Moreover, it is obvious the existence of several reticulated events that shaped the evolution of *sidJ* within *L. pneumophila*.

### Determining the influence of recombination on *sidJ* molecular evolution

**Figure 3:**
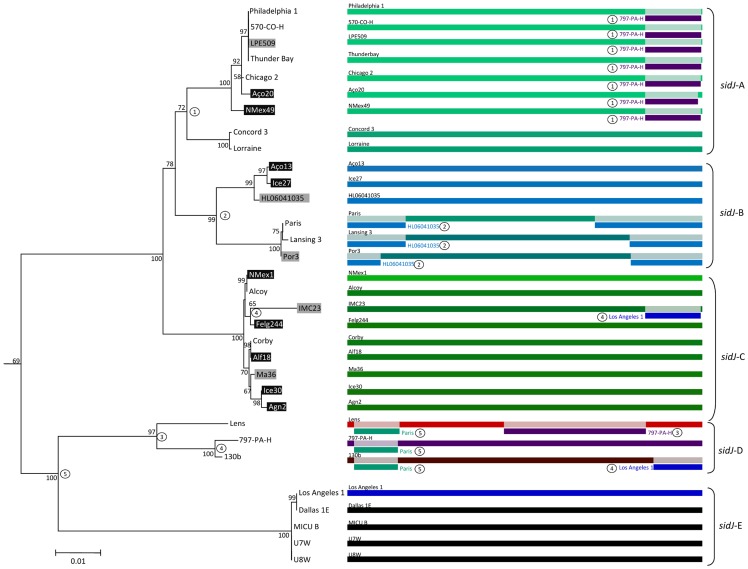
Maximum likelihood tree from *sidJ* alignment of *L. pneumophila* strains. Bootstrap support values (1,000 replicates) for nodes higher than 50% are indicated. Unique recombination events detected by six recombination detection tests implemented under the RDP3 and GARD based on *sidJ* amino acid alignment are mapped onto the corresponding breaking point positions in the alignment. Only recombination events that were identified, simultaneously, by four or more methods were selected and numbered according to the RDP analysis (see Table S3).

The aforementioned results strongly suggest the existence of recombination events between and within distinct *sidJ* subgroups. To clarify this hypothesis, evidence for individual recombination events were sought by using two approaches, RDP3 [58] and GARD [65], with only minor differences. Indeed, five putative recombinant regions were identified in this analysis and mapped onto the corresponding ML phylogenetic tree (Fig. 3 and Table S4). From it we were able to identify Potential Recombination Events (PREs) that were compatible with numerous conflicting phylogenetic signals previously observed both in the ML and Neighbor-Net analysis (Fig. 1B and 2).

The identified PREs were limited to strains belonging to the *L. pneumophila* subsp. *pneumophila* and aided to explain the previously observed complex evolutionary history of *sidJ* within this subspecies. Namely, PRE1 involving some of the strains clustered in *sidJ*-A and the ancestor *L. pneumophila* subsp. *pneumophila* strain 797-PA-H as minor parent (Fig. 3), responsible for the bifurcation denoted in the ML and Neighbor-Net analysis (Fig. 1B and 2). PRE2 involving only some strains of *sidJ*-B cluster and the ancestor *L. pneumophila* subsp. *pneumophila* strain HL06041035 as minor parent, reconstructs a previously assigned conflicting signal in the network that originated the split of the cluster into two branches (Fig. 3). Moreover, it was possible to identify PREs that helped to explain the complex evolutionary history observed within strains IMC23, Lens, 130b and 797-PA-H (PRE number 3, 4 and 5 in Fig. 3 and Table S4).

The detection of intragenic recombination events, within a gene, in opposition to intergenic recombination events, between genes, in *L. pneumophila* has been rarely reported although it is worth noticing that we have found that this form of recombination has a fundamental role on the molecular evolution of *L. pneumophila* genes critical for virulence, namely in the *dotA* gene [18] and in *sidJ* (current study). We anticipate that the reason why the impact of intragenic recombination events on the population structure and genetic diversity of *L. pneumophila* is underestimated relates with the fact that, despite the ubiquitous character of *Legionella* sp. in water environments, most studies on genetic variation in *L. pneumophila* focus on strains isolated from man-made environments, including air conditioning-systems, potable water distribution systems, public fountains, and plumbing fixtures and on clinical-related strains [14]–[16], [36], [88]–[90]. In fact those studies showed clear differences between the populations of clinical-related and man-made environmental isolates, with clinical-related isolates showing less diversity than man-made environmental isolates [14]–[16]. Recently, the first complete genome sequence of a man-made environmental *L. pneumophila* isolate was determined [31]. It was further demonstrated that this man-made environmental strain was unable to overcome the defense conferred by primary macrophages from mice known to be permissive for clinical-related *L. pneumophila* strains. Those results also suggested the existence of a host immune surveillance mechanism differing from those currently known in responding to *L. pneumophila* infection [91].

### *sidJ* gene polymorphism

Multiple alignments of the *sidJ* sequences revealed numerous substitutions, between and within the defined clusters. We further analyzed the number of polymorphic sites by using DnaSP software [54]. As a whole, the aligned sequences had 16.4% polymorphic nucleotide sites (432 of 2.628 nucleotides), 149 of which predicted amino acid replacements. SidJ length varied between 876 amino acids within cluster *sidJ*-C and 875 amino acids within the remaining clusters.

The number of polymorphic nucleotide sites detected was somewhat distinct between the defined subgroups (Table 2). The cluster *sidJ*-D was the most variable subgroup with 3.3% polymorphic sites (86/2.2625 nucleotides), 31 of which predicted amino acid replacements (36%). In contrast, the cluster *sidJ*-E was the most conserved, with only 0.1% variable sites (3/2625 nucleotides), all predicting amino acid replacements. Clusters *sidJ*-A and *sidJ*-B had 2.9% (69/2625 nucleotides) polymorphic sites, 30% and 25% of which predicted amino acid replacements, respectively. An important observation was that although only 43 of 2.2628 nucleotides were polymorphic sites (1.6%) in cluster *sidJ*-C, 70% corresponded to replacement substitutions.

**Figure 4:**
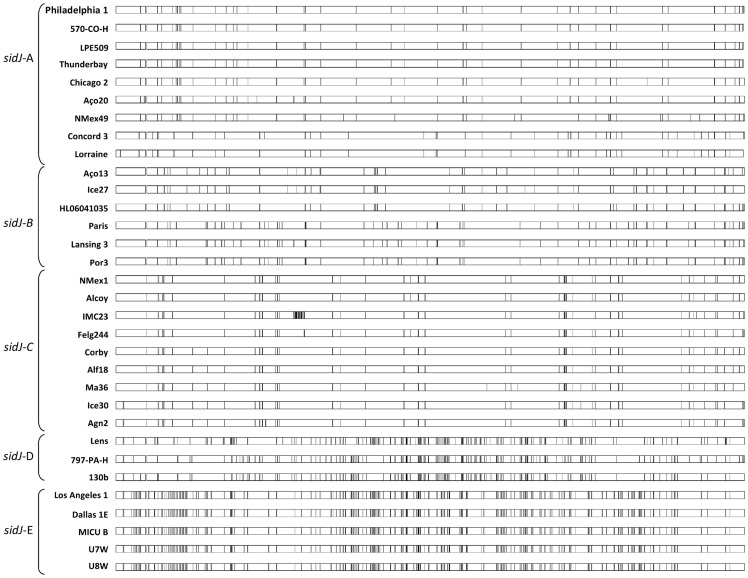
Graphical display of the location of polymorphic sites (SPNs and INDELs) of *sidJ* from *L. pneumophila* strains (see **Table 1**) using the program HAPPLOT when aligned with *L. pneumophila* strain Philadelphia 1. Polymorphic nucleotide sites based upon pairwise comparisons are represented by vertical lines. SNPs and INDELS are important drivers of bacterial evolution, by modifying how or whether gene are transcribed and translated.

In order to search for mosaic patterns, a hallmark of recombination, *sidJ* genes were aligned and the positions of sequence differences relative to a guiding sequence were visualized using the Happlot program. Numerous clusters of polymorphic sites that matched the previously identified potential recombination events in *sidJ* were readily identified by visual inspection, as shown in Fig. 4. This is a remarkable observation since obvious mosaics have only rarely been described, presumably because recombination is so effective that mosaics rapidly become too fragmented for facile recognition.

It is worth notice the degree of nucleotide polymorphisms between *sidJ* clusters A, B and C when compared with clusters *sidJ*-D and E, clearly indicating that there are several *sidJ* alleles. Additionally, *sid*-D and *sid*-E clusters were exclusively composed of clinical-related strains. Interestingly, the amino acid variations within cluster *sidJ*-E, comprising strains belonging to *L. pneumophila* subsp. *pascullei* and *fraseri*, were widely distributed throughout the gene. A similar pattern was also observed for cluster *sidJ*-D, although a cluster of polymorphic region was detected in the middle region of the gene.

### Determining the forces shaping *sidJ* sequence evolution

In order to discard any influence of positive selection in the detection of recombination events [92], we performed neutrality tests on *sidJ* gene (Table S5) and complemented them with the analysis of positively selected codons in the coding region. These tests revealed that most variation in this locus was not significantly different from the neutral hypothesis of evolution [67]–[69]. Additionally, the *sidJ* alignment was analyzed by using a codon based ML method implemented in Selecton package [76]. The server was run with the M8 model [78] and compared with the M8a null model [77]. Likelihood ratio tests between both models were not significant (cut-off value at 0.05) for *sidJ*. Therefore, the existence of positively selected codons was discarded, reinforcing the existence of recombination events.

### *sidJ* genetic context

Since *sidJ* is organized in a operon-like structure with members of the *sidE* family in several clinical-related strains [29]–[32] we considered if the same genetic structure was present in the natural and man-made environmental analyzed strains. Different primer combinations ensured that the associations between *sidJ* and the *sidE-*family members could be determined (Fig. S1 and Table S2). We have found that *sedC*, *laiE*, *sidJ*, *sedB* and *sedA* genes are structurally linked in all *L. pneumophila* examined strains, with only one likely exception, since no amplicon was obtained for the man-made environmental strain IMC23. These findings suggest that this operon-like structure has been preserved through evolution, reinforcing the relationship between *sidJ* and other members of the *sidE* family.

## Conclusions

In sum, the detection of balancing selection operating on *sidJ* evolution emerges as a clear result from various analyses performed in the present study. Furthermore, *sidJ* genetic plasticity acquired by frequent recombination events and nonsynonymous mutations is favored as a strategy in the *L. pneumophila* evolutionary adaptive process. These events are important for increasing *L. pneumophila* genetic pool by allowing the selection of new allelic forms with increase fitness or, in a more neutral perspective, as merely genetic modifications with no obvious selective advantages. Nevertheless, the detected intragenic recombination events are crucial for the increase of *sidJ* allelic diversity, contributing for the resilience of *L. pneumophila.* Further studies focusing the pathogenicity of *L. pneumophila* natural environmental strains, including the identification of virulent determinants to exploit host functions, will certainly clarify the importance of the reported polymorphism in *sidJ.*

## Supporting Information

Figure S1 **Schematic representation of the operon-like structure comprising some members of the** ***sidE*** **family, namely** ***sedC*** **,** ***laiE*** **,** ***sidJ*** **,** ***sedB*** **and** ***sedA*** **in** ***L. pneumophila*** **Philadelphia 1 (lpg2153, lpg2154, lpg2155, lpg2156 and lpg2157, respectively).** Primers used for PCR amplifications are also represented (Table S2).
(DOCX)

Table S1 **Locus tag and accession numbers from the** ***L. pneumophila*** **unrelated strains, isolated from distinct environments, type and reference strains included in this study.**
(DOCX)

Table S2 **Primers and their sequences designed in this study.**
(DOCX)

Table S3 **Genetic pairwise differences, average and standard deviation (SD) for (A) and between (B)** ***sidJ*** **and** ***rpoB*** **clusters.** The highest population pairwise differences, average and standard deviation for each gene are marked in bold.
(DOCX)

Table S4 **Potential recombinant events (PRE) identified with RDP3 from the alignment of** ***sidJ*** **obtained from 32** ***L. pneumophila*** **strains.** The minimum number of independent recombination events (IREs) within each identified PRE was inferred by a minimum of four methods and were mapped on the phylogenetic tree (Fig. 3).
(DOCX)

Table S5 D (Tajima), D* and F* (Fu and Li) and Fs (Fu) statistics obtained from ***sidJ*** **.**
(DOCX)
