# Supplementary material for: Intragenic Recombination Has a Critical Role on the Evolution of Legionella pneumophila Virulence-Related Effector sidJ
Source: PLoS One. 2014 Oct 9;9(10):e109840. doi: 10.1371/journal.pone.0109840 (PMC4192588; doi:10.1371/journal.pone.0109840)
Supplement: Figure S1 — Schematic representation of the operon-like structure comprising some members of the sidE family, namely sedC , laiE , sidJ , sedB and sedA in L. pneumophila Philadelphia 1 (lpg2153, lpg2154, lpg2155, lpg2156 and lpg2157, respectively). Primers used for PCR amplifications are also represented (Table S2). (DOCX) [file pone.0109840.s001.docx]

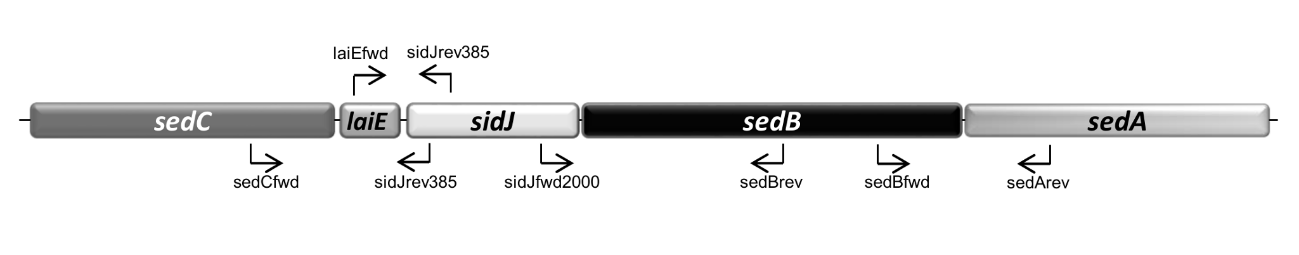


Figure S1. Schematic representation of the operon-like structure comprising some members of the *sidE* family, namely *sedC*, *laiE*, *sidJ*, *sedB* and *sedA* in *L. pneumophila* Philadelphia 1 (lpg2153, lpg2154, lpg2155, lpg2156 and lpg2157, respectively). Primers used for PCR amplifications are also represented (Table S2).
