# Supplementary material for: Intragenic Recombination Has a Critical Role on the Evolution of Legionella pneumophila Virulence-Related Effector sidJ
Source: PLoS One. 2014 Oct 9;9(10):e109840. doi: 10.1371/journal.pone.0109840 (PMC4192588; doi:10.1371/journal.pone.0109840)
Supplement: Table S1 — Locus tag and accession numbers from the L. pneumophila unrelated strains, isolated from distinct environments, type and reference strains included in this study. (DOCX) [file pone.0109840.s002.docx]

**Table S1.** Locus tag and accession numbers from the *L. pneumophila* unrelated strains, isolated from distinct environments, type and reference strains included in this study.

|  | Loci | | | | |
| --- | --- | --- | --- | --- | --- |
| Strain designation | *rpoB* | |  | *sidJ* | |
|  | Accession number | Reference |  | Accession number | Reference |
| Agn2 | FN652358 | [18] |  | HG531938 | this study |
| Felg244 | FN652410 | [18] |  | HG531941 | this study |
| Ice30 | FN652434 | [18] |  | HG531943 | this study |
| Alf 18 | FN652367 | [18] |  | HG531939 | this study |
| NMex1 | FN652468 | [18] |  | HG531947 | this study |
| NMex49 | FN652474 | [18] |  | HG531948 | this study |
| Aço20 | FN652349 | [18] |  | HG531937 | this study |
| Aço13 | FN652347 | [18] |  | HG531936 | this study |
| Ice27 | FN652433 | [18] |  | HG531942 | this study |
| Ma36 | FN652462 | [18] |  | HG531946 | this study |
| Por3 | FN652476 | [18] |  | HG531949 | this study |
| IMC23 | FN652428 | [18] |  | HG531944 | this study |
| Chicago 2 (ATCC 33215) | AY036041 | [83] |  | HG531934 | this study |
| Concord 3 (ATCC 35096) | AY036043 | [83] |  | HG531940 | this study |
| 797-PA-H (ATCC 43130) | AY036046 | [83] |  | HG531935 | this study |
| Los Angeles 1(ATCC 33156^T^) | AY036050 | [83] |  | HG531954 | this study |
| Dallas 1E (ATCC 33216) | AY036051 | [83] |  | HG531953 | this study |
| Lansing 3 (ATCC 35251) | AY036052 | [83] |  | HG531945 | this study |
| U8W (ATCC 33737^T^) | AJ746049 | [17] |  | HG531950 | this study |
| U7W (ATCC 33736) | AJ746050 | [17] |  | HG531951 | this study |
| MICU B (ATCC 33735) | AJ746051 | [17] |  | HG531952 | this study |
| 130b | LPW_04011 | [39] |  | LPW_23291 | [39] |
| Alcoy | lpa_00529 | [38] |  | lpa_03087 | [38] |
| ATCC43290 | lp12_0323 | [30] |  | lp12_2147 | [30] |
| Corby | NC_009494 | [37] |  | LPC_1604 | [37] |
| HL06041035 | LPV_0412 | [12] |  | LPV_2405 | [12] |
| Lens | lpl0362 | [36] |  | lpl2083 | [36] |
| Lorraine | LPO_0372 | [12] |  | LPO_2218 | [12] |
| LPE509 | LPE509_02910 | [31] |  | LPE509_00944 | [31] |
| Paris | lpp0387 | [36] |  | lpp2094 | [36] |
| Philadelphia 1 (ATCC 33152^T^) | AF367748 | [83] |  | lpg2155 | [83] |
| Thunder Bay | LP6_0314 | [32] |  | LP6_2184 | [32] |
