# Supplementary material for: Intragenic Recombination Has a Critical Role on the Evolution of Legionella pneumophila Virulence-Related Effector sidJ
Source: PLoS One. 2014 Oct 9;9(10):e109840. doi: 10.1371/journal.pone.0109840 (PMC4192588; doi:10.1371/journal.pone.0109840)
Supplement: Table S2 — Primers and their sequences designed in this study. (DOCX) [file pone.0109840.s003.docx]

**Table S2** - Primers and their sequences designed in this study.

| **Primer** | **Gene** | **Locus tag** | **Sequence** | **Location^b^** | **Aplication^c^** |
| --- | --- | --- | --- | --- | --- |
| sidJF | *sidJ* | lpg2155 | 5’-ATGTTTGGTTTCATAAAGAAAGT-3’ | 1-20 | A, S |
| sidJR | *sidJ* | lpg2155 | 5’-TTATWKCAARCGTTTATCAGWRKTAC-3’ | 2603-2628 | A,S |
| sidJF2 | *sidJ* | lpg2155 | 5’-CAAAGCGCCTTTRTACAAAA-3’ | 763-783 | S |
| sidJR2 | *sidJ* | lpg2155 | 5’-CCRAGYGTTAACTGAATAACC-3’ | 1827-1848 | S |
| sidJF3^a^ | *sidJ* | lpg2155 | 5’-GATGGTGTTCATCAAGACC-3’ | 1625-1645 | S |
| sdeBrev | *sedB* | lpg2156 | 5’-AACCAGTGATAACAAAATATC-3’ | 2928-2948 | A, S |
| sidJfwd2000 | *sidJ* | lpg2155 | 5’-CTAATATTGAAAAACATTTC-3’ | 2006-2025 | A, S |
| sidJrev385 | *sidJ* | lpg2155 | 5’-TCTATCCCATTAAAAGAAATTCCTT-3’ | 385-406 | A, S |
| laiEfwd | *laiE* | lpg2154 | 5’-ATCATCTGTATGGCGCTAAACA-3’ | 190-211 | A, S |
| sedCfwd | *sedC* | lpg2153 | 5’-AAACGAGTCACCGACTTTGA-3’ | 3421-3440 | A, S |
| sedArev | *sedA* | lpg2157 | 5’-TCCATGCAACGCTGGATAGG-3’ | 1303-1322 | A, S |
| sedBfwd | *sedB* | lpg2156 | 5’-GCAAAAGCTACTCCAATAGAT-3’ | 4441-4461 | A, S |
|  |  |  |  |  |  |
|  |  |  |  |  |  |

^a^ Primers used for sequencing *L. pneumophila* subsp. *fraseri* (SGs 4, 5, and 15)

^b^ Locations in the aligned data set of nucleotide sequences from *L. pneumophila* Philadelphia 1

^c^ A, amplification; S, sequencing.
