# Supplementary material for: Intragenic Recombination Has a Critical Role on the Evolution of Legionella pneumophila Virulence-Related Effector sidJ
Source: PLoS One. 2014 Oct 9;9(10):e109840. doi: 10.1371/journal.pone.0109840 (PMC4192588; doi:10.1371/journal.pone.0109840)
Supplement: Table S3 — Genetic pairwise differences, average and standard deviation (SD) for (A) and between (B) sidJ and rpoB clusters. The highest population pairwise differences, average and standard deviation for each gene are marked in bold. (DOCX) [file pone.0109840.s004.docx]

**Table S3.** Genetic pairwise differences, average and standard deviation (SD) for (A) and between (B) *sidJ* and *rpoB* clusters. The highest population pairwise differences, average and standard deviation for each gene are marked in bold.

| A. |  |  |  |  |  |  |  |  |  |  |  |
| --- | --- | --- | --- | --- | --- | --- | --- | --- | --- | --- | --- |
|  | *sidJ* | | | | | |  | *rpoB* | | | |
|  |  | clusters | | | | Overall |  | clusters | |  | Overall |
|  | A | B | C | D | E |  |  | A | B | C |  |
| Pairwise differences | 0-0.022 | 0-0.026 | 0-0.013 | 0.006-0.014 | 0-0.002 | **0-0.070** |  | 0-0.010 | 0-0.010 | 0 | 0-0.032 |
| Diversity Average (SD) | 0.010 (0.002) | 0.015 (0.003) | 0.004 (0.001) | 0.011 (0.003) | 0.001 (0.001) | 0.033 (0.003) |  | 0.004 (0.004) | 0.007 (0.006) | 0 | **0.043 (0.006)** |

| B. |  |  |  |  |  |
| --- | --- | --- | --- | --- | --- |
| Diversity average (SD) | | | | | |
|  | *sidJ* clusters | | | | |
|  | A | B | C | D | E |
| A |  | 0.032 (0.006) | 0.035 (0.007) | 0.075 (0.014) | 0.090 (0.016) |
| B |  |  | 0.040 (0.007) | 0.080 (0.014) | **0.093 (0.016)** |
| C |  |  |  | 0.078 (0.014) | 0.089 (0.016) |
| D |  |  |  |  | 0.074 (0.013) |
|  | *rpoB* clusters | | |  |  |
|  | A | B | C |  |  |
| A |  | **0.017 (0.012)** | 0.013 (0.011) |  |  |
| B |  |  | 0.003 (0.003) |  |  |
