# Supplementary material for: Intragenic Recombination Has a Critical Role on the Evolution of Legionella pneumophila Virulence-Related Effector sidJ
Source: PLoS One. 2014 Oct 9;9(10):e109840. doi: 10.1371/journal.pone.0109840 (PMC4192588; doi:10.1371/journal.pone.0109840)
Supplement: Table S4 — Potential recombinant events (PRE) identified with RDP3 from the alignment of sidJ obtained from 32 L. pneumophila strains. The minimum number of independent recombination events (IREs) within each identified PRE was inferred by a minimum of four methods and were mapped on the phylogenetic tree (Fig. 3). (DOCX) [file pone.0109840.s005.docx]

**Table S4.** Potential recombinant events (PRE) identified with RDP3 from the alignment of *sidJ* obtained from 32 *L. pneumophila* strains. The minimum number of independent recombination events (IREs) within each identified PRE was inferred by a minimum of four methods and were mapped on the phylogenetic tree (Fig. 3).

| **Recombinational** | **Breakpoints** | **Minimum number** | ***p*-Val** | **Recombination detection tests** | | | | | |
| --- | --- | --- | --- | --- | --- | --- | --- | --- | --- |
| **events** |  | **of IREs per PRE** |  | **RDP** | **GENECONV** | **MaxChi** | **Chimaera** | **SiScan** | **Bootscan** |
| 1 | 2205-2615 | 7 | 1.8 x 10^-4^ | 7 | 5 | 7 | 7 | 7 | 4 |
|  | 2205-2597 |  |  |  |  |  |  |  |  |
| 2 | 1833-431 | 3 | 4.9 x 10^-5^ | - | - | 3 | 3 | 3 | 3 |
|  | 2092-430 |  |  |  |  |  |  |  |  |
|  | 2098-248 |  |  |  |  |  |  |  |  |
| 3 | 2205-2614 | 2 | 6.9 x 10^-2^ | 2 | 1 | 2 | 2 | 1 | 2 |
|  | 2267-2628 |  |  |  |  |  |  |  |  |
| 4 | 1157-2211 | 1 | 4.5 x 10^-21^ | 1 | 1 | 1 | 1 | 1 | 1 |
| 5 | 49-385 | 3 | 2.1 x 10^-8^ | 3 | 3 | 3 | 3 | 3 | 3 |
|  | 49-375 |  |  |  |  |  |  |  |  |
