# Supplementary material for: Intragenic Recombination Has a Critical Role on the Evolution of Legionella pneumophila Virulence-Related Effector sidJ
Source: PLoS One. 2014 Oct 9;9(10):e109840. doi: 10.1371/journal.pone.0109840 (PMC4192588; doi:10.1371/journal.pone.0109840)
Supplement: Table S5 — D (Tajima), D* and F* (Fu and Li) and Fs (Fu) statistics obtained from sidJ . (DOCX) [file pone.0109840.s006.docx]

**Table S5.** D (Tajima), D* and F* (Fu and Li) and Fs (Fu) statistics obtained from *sidJ*.

| Locus | D | D* | F* | Fs |  |
| --- | --- | --- | --- | --- | --- |
| *sidJ* | 0.315 | 1.04 | 0.94 | 6.91 | Neutral |
